# Closed spinal dysraphism-lipomyelocele in an 18-year-old male

**DOI:** 10.11604/pamj.2022.42.60.33893

**Published:** 2022-05-23

**Authors:** Nikita Hitesh Seth, Ragini Dadgal

**Affiliations:** 1 Ravi Nair Physiotherapy College, Datta Meghe Institute of Medical Sciences, Sawangi, Wardha, Maharashtra, India

**Keywords:** Lipomyelocele, physical therapy, unhealing foot ulcers

## Image in medicine

We are reporting a magnetic resonance imaging (MRI) finding of an 18-year-old male not a known case of any chronic medical illness presented with chief complaints of swelling over the lumbosacral region since birth with sensory loss over bilateral lower limb for 2 months. He also complained of unhealing ulcers over the dorsum of the great toe. Magnetic Resonance Imaging (MRI) findings revealed T2 hyperintensive mass (red arrow) of subcutaneous soft tissue in the posterior lumbosacral region extending into the spinal canal. Posterior spinal defect at the L3-L4 level with tethering of cord was noted, suggestive of Lipomyelocele. Following the removal of the Lipomyelocele and detethering of the chord, the patient suffered bladder dysfunction and right foot drop. For additional treatment, he was referred to a physiotherapist. He had weakness in his lower limbs (Medical Research Council (MRC) grade 3), a foot drop on the right side, and considerable muscular loss on physical examination. Sensations were affected on both sides. Bladder training, sensory re-education, lower limb muscular strengthening, and foot drop management were all part of the physiotherapy rehabilitation. Foot drop was treated with an ankle-foot orthosis and dorsiflexion reflex stimulation, with an emphasis on activities in a weight-bearing position. It has been discovered that a multidisciplinary strategy involving medical, surgical, and physical therapy improves treatment outcomes.

**Figure 1:**
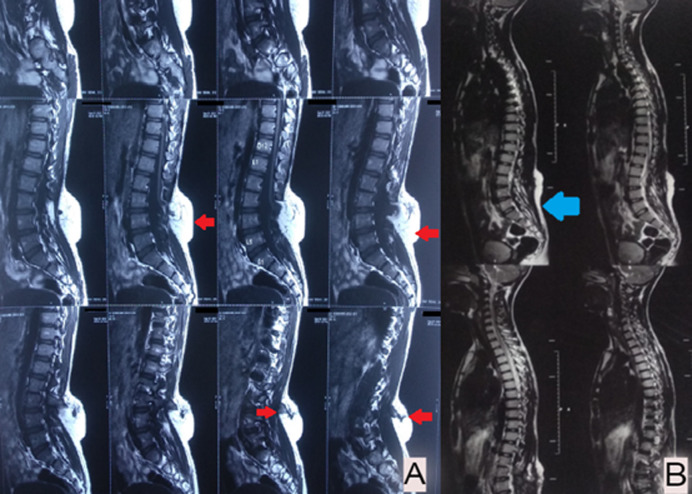
A) red arrow indicates lipomyelocele at lumbosacral region; B) post-operative MRI, blue arrow indicates thickened filum terminale and tethering at L2-L3 level

